# Potential Factors Influencing the Effects of Anthocyanins on Blood Pressure Regulation in Humans: A Review

**DOI:** 10.3390/nu11061431

**Published:** 2019-06-25

**Authors:** Stefano Vendrame, Dorothy Klimis-Zacas

**Affiliations:** School of Food and Agriculture, University of Maine, Orono, ME 04469, USA; stefano.vendrame@fulbrightmail.org

**Keywords:** anthocyanins, hypertension, blood pressure, berries

## Abstract

Dietary intake of anthocyanins (ACNs) is associated with a reduced risk of cardiovascular and coronary heart disease. While the anti-inflammatory, antioxidant, and lipid-lowering effects of ACN consumption have been consistently reported, their effect(s) on blood pressure regulation is less consistent and results from human studies are mixed. The objective of this review is attempting to identify potential patterns which may explain the variability in results related to blood pressure. To do so, we review 66 human intervention trials testing the effects on blood pressure of purified ACN or ACN-rich extracts, or whole berries, berry juices, powders, purees and whole phenolic extracts, from berries that are rich in ACN and have ACNs as predominant bioactives. Several factors appear to be involved on the mixed results reported. In particular, the baseline characteristics of the population in terms of blood pressure and total flavonoid intake, the dose and duration of the intervention, the differential effects of individual ACN and their synergistic effects with other phytochemicals, the ACN content and bioavailability from the food matrix, and individual differences in ACN absorption and metabolism related to genotype and microbiota enterotypes.

## 1. Introduction

Hypertension is a known risk factor of cardiovascular disease (CVD), which is the leading cause of death worldwide [1]. The role of lifestyle modification, including diet, as a means to reduce CVD risk is attracting increasing interest, but requires a careful evidence-based approach [2]. This is especially true for management and control of hypertension, which burdens most European and Western countries [3], 

Anthocyanins (ACN) are a large class of water-soluble plant metabolites belonging to the flavonoid family of phenolics, responsible for the red, blue, and purple pigmentation of many flowers, fruits, and vegetables. They are the glycosylated forms of highly reactive and relatively unstable molecules characterized by a flavylium cation structure, called anthocyanidins [4]. Although more than 600 naturally occurring ACNs have been identified, for the vast majority, they are made by different glycosylated forms of only six anthocyanidins: pelargonidin, cyanidin, delphinidin, peonidin, petunidin, and malvidin [5]. The average daily intake of ACNs is 200 mg/day, which is about one fifth of the average daily intake of total phenolics [6]. While plants produce ACN as a defense against environmental stressors, such as temperature extremes, UV light, and drought, dietary intake of anthocyanins has been extensively studied for its health-promoting potential, in particular with respect to cardiovascular disease prevention [7].

Strong epidemiological evidence associates ACN intake with a reduced risk for cardiovascular disease and coronary heart disease [8]. While ACN consumption has been consistently associated with anti-inflammatory, antioxidant, and lipid-lowering effects, its effect on blood pressure regulation is less consistent and results from human studies are mixed [9,10].

To try to identify potential patterns explaining such variability in results related to blood pressure, we decided to review clinical trials testing purified ACN or ACN-rich extracts, or whole berries, berry juices, powders, purees, or whole phenolic extracts from berries that are rich in ACNs and have ACNs as predominant bioactives. Among berries, a higher ACN content can be found in chokeberries (*Aronia melanocarpa*) (over 400 mg/100 g) [11], blackcurrants, blueberries, and raspberries (over 100 mg/100 g), followed by blackberries and cherries (over 50 mg/100 g) [12,13]. Instead, cranberries, grapes, and strawberries contain, on average, less than 50 mg/100 g, and more of other bioactive phytochemicals [6,14]. Thus we chose not to consider them in this review as a proxy for ACNs, unless they were used as part of berry mixes or as a source of ACN extracts. 

## 2. ACNs and Blood Pressure

High blood pressure can be caused by an increased cardiac output or by a higher systemic vascular resistance, which in turn results either from a narrower diameter of blood vessels or by their lower elasticity. An excess of vasoconstricting stimuli, or a lack of vasodilator stimuli, can both result in hypertension [1]. ACNs have been associated with blood pressure regulation, directly or indirectly, by three major mechanisms:

1. ACNs have been consistently shown to increase endothelial-derived nitric oxide (NO), via modulation of endothelial NO synthase (eNOS) expression and activity. Nitric oxide is one of the major contributors to endothelium-dependent vasorelaxation. It causes vascular smooth muscle relaxation following activation of soluble guanylate cyclase, which in turn increases cGMP. This blocks the release of intracellular calcium, preventing it from causing vascular smooth muscle contraction [15].
2. Reactive oxygen species damage NO, thus promoting vasoconstriction and hypertension. Due to their strong antioxidant activity, ACNs act to prevent NO oxidative damage and radical-induced NO conversion, such as the reaction caused by NADPH oxidase [16].
3. ACNs have been shown to reduce synthesis of vasoconstricting molecules, such as angiotensin II via inhibition of the angiotensin-converting enzyme (ACE) activity, endothelin-1, and thromboxanes via inhibition of the cyclooxygenase (COX) pathway [17].

While most of these mechanistic observations come from in vitro and animal model studies [18], the practical outcome of ACN dietary consumption on blood pressure regulation in humans is likely complicated by multiple factors.

### 2.1. Epidemiological and Meta-Analysis Data

Epidemiological data appears to confirm the existence of a link between ACNs and blood pressure regulation. A prospective epidemiological study on 34,489 postmenopausal women from the Iowa Women’s Health Study, analyzing the effects of total flavonoids or seven different individual flavonoid subclasses on cardiovascular health over 12 years, found a significant inverse association between ACNs and CHD and CVD and total mortality, while total flavonoids and the other individual subclasses had no significant effect [6]. Analyzing data from a cohort of 156,957 men and women from the Nurses’ Health Study, the NHS I and the Health Professionals Follow-Up Study, followed for 14 years, Cassidy et al. found an inverse association between ACN intake and hypertension [19]. Interestingly, this association was not observed for total flavonoid intake, nor for any other subclass of flavonoids (flavones, flavonols, flavan-3-ols, and flavanones), with the exception of two single compounds (apigenin and catechin) [19]. In a cross-sectional study from a cohort of 1898 adult women from the TwinsUK registry, a higher intake of ACNs was associated with significantly lower central systolic blood pressure (SBP) and mean arterial pressure (MAP). Again, the inverse association was not observed for total flavonoid intake, flavanones, flavan-3-ols, flavonols, or flavones [20].

Several meta-analyses have been conducted using data from clinical trials involving sources of ACNs, with mixed results. A meta-analysis of 128 clinical trials on different sources of ACN and ellagitannins, with a total of 5538 participants, found that both systolic and diastolic blood pressure (DBP) were significantly lowered by consumption of berries, red grapes, and red wine—the main sources of ACN investigated in the study [12]. The effect is also significant when studies on berries only and studies on red wine/red grapes only are considered [12]. A meta-analysis of 22 clinical trials studying the effects of berries (total 1251 subjects), found a significant reduction of SBP, but not DPB [21]. Per contra, a meta-analysis on 32 clinical trials (1491 total participants) investigating the effects of ACNs and ACN sources on cardiometabolic health, found that the reduction in SBP and DBP did not reach statistical significance [22]. Similarly, a meta-analysis of 19 clinical studies found no significant effect of ACN supplementation on either SB or DBP [23]. A meta-analysis of six clinical studies with 472 total participants, found no significant effect of ACN supplementation on either SBP or DBP [24]. A meta-analysis of six clinical trials with 204 total participants, detected no significant effect of blueberry consumption on blood pressure [25]. The participants of all the above mentioned meta-analyses represented a mixed population, including men and women of all age groups and from different geographical regions, both healthy and with cardiovascular risk factors. 

In light of such mixed results, we searched the literature for clinical trials, testing the effects on blood pressure of ACNs or ACN-rich berries and examined the results individually to attempt to identify potential patterns explaining the variability in results related to blood pressure.

### 2.2. Literature Search

**Figure 1:**
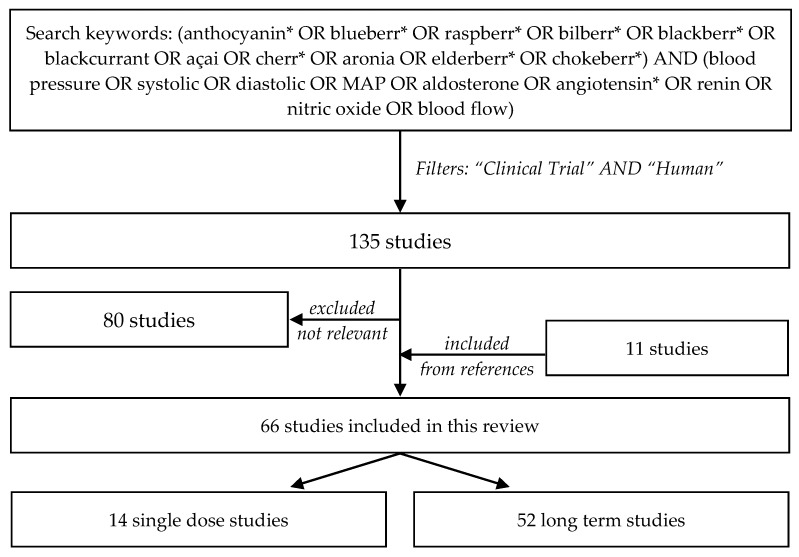
Flow chart describing the literature search process. The literature search was conducted on PubMed, ScienceDirect, and Web of Science databases and limited to original clinical trials with human participants published after January 1st, 2005. One-hundred-and-thrity-five abstracts and full texts were reviewed. Eighty studies not reporting both ACN source and blood pressure markers were excluded. Eleven additional relevant studies were identified during the screening process from the reference lists, and were added to the review.

A search of the literature for human acute and chronic intervention studies was carried out on multiple databases (PubMed, ScienceDirect, and Web of Science) with the keywords (anthocyanin* OR blueberr* OR raspberr* OR bilberr* OR blackberr* OR blackcurrant OR açai OR cherr* OR aronia OR elderberr* OR chokeberr*) AND (blood pressure OR systolic OR diastolic OR MAP OR aldosterone OR angiotensin* OR renin OR nitric oxide OR blood flow). Abstracts and full texts were screened, and reference lists were also searched for related articles. Further details about the literature search process are provided in Figure 1. Sixty-six relevant studies were identified [26,27,28,29,30,31,32,33,34,35,36,37,38,39,40,41,42,43,44,45,46,47,48,49,50,51,52,53,54,55,56,57,58,59,60,61,62,63,64,65,66,67,68,69,70,71,72,73,74,75,76,77,78,79,80,81,82,83,84,85,86,87,88], and their results related to blood pressure are summarized in Table 1 and Table 2.

### 2.3. Single-Dose Interventions

Of the 14 single-dose interventions which were identified and reviewed; six found a significant effect on blood pressure, as summarized in Table 1.

All single-dose studies were performed on healthy participants, with the exception of the study by Keane et al. which was conducted on 15 subjects with early hypertension: both SBP and mean arterial pressure, but not DBP, were significantly lower at 1, 2, 3, 4, 5, 6, 7, and 8 hours after a serving of tart cherry juice providing 73.5 mg ACN [31]. Del Bo et al. investigated the effect of a serving of blueberry juice providing 300–350 mg ACN in restoring blood pressure after a cigarette was smoked by young otherwise healthy smokers. In one study, the SBP spike induced by smoke was restored by the blueberry juice [28], but the effect was not replicated in a following study [29].

### 2.4. Long-Term Interventions

Fifty-two long term interventions were identified and summarized in Table 2. For obvious practical reasons, and for the purpose of standardization, only few studies used fresh berries, while most studies used more stable, but processed berries such as juices, concentrates, or freeze-dried powders. Eleven studies used only phenolic extracts, and 14 studies used isolated ACNs or ACN-rich extracts. Of the 52 reviewed interventions, 21 found a significant effect on blood pressure (Table 2).

## 3. Potential Factors Influencing ACN Effects on Blood Pressure

Several factors appear to influence the effects of ACNs on blood pressure regulation in humans, and they are discussed in the following subsections.

### 3.1. Baseline Characteristics of the Population

A consistent observation is that the effect on blood pressure is only detectable in subjects with high blood pressure baseline value. Of the six studies targeting specifically prehypertensive or hypertensive subjects, five found an effect on blood pressure [48,53,58,67,83], and only one did not [52]. In contrast, of the 16 studies enrolling completely healthy subjects with normal blood pressure, only one [68] found an effect on blood pressure, while the remaining 15 studies did not find any effect (Table 2). 

After a six-week intervention with high bush blueberries on 25 adults, McAnulty et al. did not find a significant effect on DBP for the whole group, but the reduction became significant when considering only the subset of nine prehypertensive subjects [70]. Similarly, after an eight-week intervention with a mix of berries providing 515 mg ACNs, on 72 subjects with CVD risk factors, Erlund et al. found an overall significant reduction in SBP, but observed that the effect was very strong in the subset of hypertensive participants [49]. Following 12 weeks consumption of a mixed berry fruit juice, a group of 134 prehypertensive or hypertensive subjects had a significant reduction in SBP, but the reduction was more pronounced in the subset of participants with higher baseline blood pressure values [83].

Another interesting observation comes from the study of Cook et al. on 13 healthy participants receiving a blackcurrant extract providing 315 mg ACNs for a week [44]. While no effect on blood pressure was observed at rest, a significant reduction in SBP, DBP, and MAP was observed during isometric contraction, suggesting once more that ACNs act to lower BP when it is higher than normal, but do not lower it when it is already in a healthy range [44].

Based on these findings, future studies should aim to identify with more precision which population segments may benefit from ACN intake, possibly relating the findings to the new American Heart Association guidelines’ blood pressure categories of normal (<120/<80 mmHg), elevated (120–129/<80 mmHg), stage 1 hypertension (130–139/80–89), stage 2 hypertension (≥140/≥90 mmHg), and hypertensive crisis (>180/>120 mmHg).

Another potentially relevant factor is the baseline total flavonoid intake of the population under study. It is reasonable to hypothesize that the effect of ACN supplementation may be greater in subjects with low baseline flavonoid intake. Unfortunately, this information is rarely collected or reported in studies, making it difficult to identify a pattern.

### 3.2. Dose Effect

Of the seven studies providing high doses of ACN (>500 mg/day), four studies found a significant effect on blood pressure [38,40,49,67], while the other three studies did not find any significant effect [63,80,81]. In contrast, a significant blood pressure-lowering effect was detected by three studies providing low doses of ACN (<100 mg/day) [56,71,79].

It is important to consider, however, that ACN quantification is rather complicated, and only few studies rely on complete ACN profiles of the food under investigation. More frequently, studies measure cyanidin-3-glycoside equivalents, or rely on a quick but approximate spectrophotometric quantification following methanol extraction. In some cases, they only estimate ACN content based on the USDA food composition tables. To further complicate matters, ACNs are rather unstable compounds and sensitive to pH variations, heat, light, oxygen exposure, and enzyme activity, and they are highly reactive with other molecules, sugars, proteins, and other phenolics [4]. Thus, the food matrix, its chemical composition, the manufacturing process, storage conditions, and duration, may all significantly affect ACN content and activity at the time of consumption even when the initial quantification is accurate [89,90,91]. Indeed, when Rodriguez-Mateos et al. tested both a freeze-dried blueberry drink and a blueberry baked product prepared with the same amount of blueberry powder, the ACN content of the drink was 339 mg, while only 196 mg ACN was found in the baked blueberry product; the rest being converted to other phenolics [37]. Thus, the reported ACN content from different studies may very easily be under- or overestimated, making it extremely difficult to compare studies.

Only seven studies investigated specifically the effect of different ACN doses, of which four seem to suggest the existence of a dose-effect. After 12 days consumption of a blackcurrant extract providing either 105, 210, or 315 mg/day of ACNs, with a crossover design, a significant reduction in mean arterial pressure in a group of 15 athletes was only observed with the two higher ACN doses [43]. After an 8-week intervention with 1.5 or 2.5 g a day of black raspberry powder given to 45 prehypertensive subjects, a significant reduction of SBP was only observed in the group receiving the higher raspberry dose [53]. In a 6-week intervention with 122 older adults, Whyte et al. investigated the effects of either 1 or 2 daily grams of whole wild blueberry powder, or a 200 mg wild blueberry extract, providing 2.7, 5.4, or 14 mg ACNs, respectively. A significant reduction of SBP was only observed with the extract providing the higher ACN dose, but not with the whole berry powder [85].

In contrast, in another 6-week intervention with 66 mostly overweight adults, neither a low nor a higher dose blackcurrant juice (providing either 40 mg or 143 mg ACN daily, respectively) had any effect on blood pressure [62]. An intervention of 12 weeks on 134 prehypertensive or hypertensive subjects tested a mixed berry fruit juice providing 43 mg ACNs, or the same juice enriched with blackcurrant press residues providing 210 mg ACNs, and both juices significantly lowered SBP with no dose-dependency effect [83]. When 23 healthy participants received a single dose black-currant extract drink containing 150, 300, or 600 mg ACNs following a high-carbohydrate meal, no significant changes in blood pressure were observed for any of the doses, two hours after the challenge [27]. 

Another interesting observation comes from the study of Kent et al. providing a single dose of cherry juice containing 207 mg ACNs to a group of young or older adults and detecting a significant reduction of both SBP and DBP at two hours after consumption. However, when the same amount of juice was split into three doses provided one hour apart over two hours, the effect on blood pressure was no longer detectable [34]. 

Thus, a positive effect on blood pressure was found across a wide range of ACN doses, with both dose and time being relevant factors.

### 3.3. Study Duration

The study duration does not seem a consistent factor in determining the effect of ACNs on blood pressure. Of the 34 studies with 6 week durations or longer, 15 found a significant effect on blood pressure (Table 2). Of the 19 studies with durations of 4 weeks or less, seven studies found an effect (Table 2). Thus, a blood pressure-lowering effect is found both in longer and shorter studies. Indeed, a significant effect on blood pressure can be found also following single-dose interventions (Table 1).

It is interesting to note, however, that after 6-week consumption of aronia juice providing 90 mg/day ACNs, a group of 58 male with mild hypercholesterolemia had a significant reduction in DBP but not in SBP, while after another 6 weeks of aronia juice consumption the reduction became significant also in SBP, suggesting that the duration of the intervention may indeed be a relevant factor [78].

### 3.4. Systolic vs. Diastolic Blood Pressure

Of the 28 studies registering a significant effect of blood pressure, 14 studies reported a reduction in SBP but not in DBP (Table 2), and 12 studies reported a reduction in both SBP and DBP (Table 2). Only one study found an effect on DBP but not in SBP, following a 16 weeks consumption of cold-pressed aronia juice and oven-dried aronia powder, providing 1024 mg/day of ACNs, to 37 subjects with mild hypertension [67].

Thus, the blood-pressure-lowering effect appears to be more evident on SBP than DBP.

### 3.5. Effect on Angiotensin-Converting Enzyme (ACE)

Hormonal long-term regulation of blood pressure, mainly via the renin-angiotensin-aldosterone and antidiuretic hormone (ADH) systems, is a potential target of ACN activity, as has been suggested by investigations in the animal model [92]. In humans, two studies have measured the effects of ACN consumption on the ACE.

A group of 23 subjects with untreated metabolic syndrome (MetS) received either aronia extract supplements providing 60 mg ACNs, or ACE-inhibitors. After 8 weeks, the activity of SBP, DBP, and ACE was significantly lower compared to baseline. However, ACE activity was still higher compared to a reference group of healthy controls or MetS controls treated with ACE-inhibitors (reference group was measured only once and did not undertake any intervention) [79]. Conversely, following 3-week consumption of 250 g fresh blueberries, a group of 20 overweight smokers experienced no effect on blood pressure or ACE activity [36].

More studies on how ACN intake may affect blood pressure hormonal regulatory systems are warranted, and the variability in biological responses should also be considered in view of the recent findings on the M235T polymorphism of the angiotensinogen gene, which has been linked with cardiovascular disease [93].

### 3.6. Synergistic Effects

Many studies which found significant effects on BP used ACN-rich foods or whole extracts, which also contain other bioactive phytochemicals potentially affecting blood pressure. The exact composition of such extract is often unknown in most studies. Of the 14 studies using isolated ACNs or ACN-rich extracts, only one found a significant effect on blood pressure, while the remaining 13 studies did not find any effect. In contrast, of the 11 studies using whole phenolic extracts, eight studies found a significant effect, while only three studies did not find any effect on blood pressure (see Table 2).

It is also interesting to report that while no effect on blood pressure was observed when whole grapes were given for 4 weeks to a group of 60 mildly hypertensive participants, a significant reduction of both SBP and DBP was detected with grape wine extract that had a comparable total phenolic content [48]. Thus, berry phenolics—but not isolated ACNs—appear to be effective on blood pressure, suggesting that the effect is synergistic with other molecules. Of course, it is also possible that the effect is entirely exerted by other phenolic compounds, or other phytonutrients, independent of ACNs, although as far as we know ACNs are the only molecules which are abundant and transversally present in all the different berries examined in this review, and for which positive effects on BP have been detected.

Furthermore, it is also to be noted that most of the studies testing isolated ACNs used the same commercially available supplement, made with ACNs isolated from bilberry and blackcurrant.

A synergistic effect with lifestyle and diet is also a likely relevant factor. Gurrola Diaz et al. tested the effect of 4-week consumption of a *Hibiscus sabdariffa* (HS) extract powder providing 19 mg ACN on a group of 71 healthy and 51 MetS patients, using a preventive diet as control. No significant effect on blood pressure was found either with HS alone or with diet alone. However, in the group receiving both the HS powder and the preventive diet, a significant reduction of both SBP and DBP was observed [50].

### 3.7. Differential Effect of Individual ACNs

Most interventions use fresh berries or berry extracts, which contain a mix of different compounds including different ACNs. This is undoubtedly the best approach in view of extrapolating the results to a real-life situation, in which whole foods are consumed and not isolated compounds, and the consumption of whole foods rather than supplements should be encouraged to promote health and prevent disease. However, this approach makes it more difficult to identify potential differential effects among different molecules, especially when trying to elucidate their mechanisms of action.

Indeed, when studying single anthocyanins, Rechner & Kroner observed that the inhibitory effect on the redox-sensitive p38 MAPK and c-jun-N-terminal kinase pathways often reported for ACNs, was only caused by delphinidin and cyanidin, but not malvidin and peonidin, suggesting that the hydroxyl residue in position 3 of the B ring may play a key functional role [94]. Thus, it is not unreasonable to hypothesize that the variability in the results between different studies is also at least in part due to the fact that some ACNs have a stronger effect on BP than others.

The different ACN profile of individual food sources is also a factor of variability. For example, blueberries contain predominantly delphinidin, malvidin, and petunidin; raspberries predominantly cyanidin and pelargonidin; and blackberries predominantly cyanidin and malvidin [14]. It must be noted, however, that significant effects on blood pressure have been observed with all different berries, including chokeberries, blueberries, raspberries, cherries, and blackcurrants (Table 1 and Table 2).

### 3.8. ACN Absorption and Metabolism

ACN metabolism is complex and mostly unknown, and in order to fully understand their biological functions, it is necessary to better elucidate their metabolic fate [90]. While most in vitro studies focus on isolated ACNs, it is important to remember that only less than 1% of total dietary ACNs are absorbed intact. A higher proportion of dietary ACNs is absorbed after hydrolysis and partial degradation to other phenolic compounds. Part of the unabsorbed ACNs is also fermented by the colonic microbiota, and their catabolic products are subsequently absorbed into the bloodstream [95]. Furthermore, ACNs are quite unstable at neutral pH, and after they are absorbed, ACN parent compounds, degradation products and microbial metabolites all undergo significant metabolism by both phase I and phase II enzymes to form methyl, glucuronide, and sulfate conjugated metabolites [12].

Thus, it is not enough to study the biological effects of parent ACNs, but also the effects of their numerous catabolic products based on microbiota, physiology, and health of the GI in individuals. This likely explains most of the discrepancies between the mechanisms of action suggested in vitro and the actual in vivo outcomes.

### 3.9. Interaction with Gut Microbiota

Increasing evidence links the composition of gut microbiota to key physiological effects related to the prevention of chronic disease, and this includes the contribution of gut bacteria to blood pressure regulation [96,97]. The relationship between ACNs and gut bacteria goes both ways: on one hand, ACN intake influences the composition of gut microbiome, and on the other hand, colonic fermentations transform unabsorbed ACNs to different catabolic products that can be absorbed and act as bioactives [98]. Microbial catabolism of ACNs consists mainly in the cleavage of their heterocyclic flavylium ring (the C-ring), and subsequent dehydroxylation or decarboxylation to form phenolic acids [97,99].

Thus, interaction with gut microbiome is likely an important element of variability that could explain some of the different effects observed for ACN intake. To our knowledge, however, no study has directly investigated the relationship between gut microbiota and the effects of ACN intake on blood pressure regulation.

## 4. Conclusions

A consistent number of studies documented a significant blood-pressure-lowering activity related to ACNs and ACN-rich berry consumption, suggesting that an effect indeed exists.

The fact that many other studies failed to observe such an effect, indicates that the outcome is not generalized and likely depends on many other factors, and, in particular, the baseline characteristics of the population (more specifically, their baseline blood pressure and total flavonoid intake), ACN dose, duration of the intervention, differential effects of individual ACNs, and synergistic effects with other phenolics and bioactive phytochemicals in general. Additionally, ACN content and bioavailability from the food matrix (whole food, juice, freeze-dried powder, and extract), modified by the manufacturing process and storage conditions and duration, need to be taken into account. Finally, ACN absorption and metabolism, which is affected by the different microbiota enterotypes of each individual, his/her different genotype, the physiological condition of the gastrointestinal tract and its relative response to dietary bioactives, is also a factor to be considered. 

Further research will need to identify more precisely the clinical conditions and the characteristics of individuals for which an increased consumption of ACN-rich foods may be especially recommended and could potentially reduce the dose and/or the administration of antihypertensive medications.

## Figures and Tables

**Table 1:** Summary of single dose interventions.

Reference	Study Design	Control	Participants ^a^	Source of ACN	Daily Dose of ACN ^b^	Effect on Blood Pressure ^c^
Alquarashi [26]	Crossover	Double-blind, controlled	23 healthy male, age 46 ± 9, BMI 28 ± 2	Açai-based smoothie, (control: macronutrient-matched smoothie), following high-fat meal	493 mg	= SBP, =DBP at 2 and 6 h
Castro-Acosta [27]	Crossover	Double-blind	23 healthy, age 46 ± 14, BMI 26 ± 3.8	Blackcurrant extract drink, following high-carbohydrate meal	150, 300, or 600 mg ACN	= SBP, =DBP after 2 h
Del Bo [28]	Crossover	Controlled	10 younger adults, age 21 ± 2, BMI 23 ± 2	Blueberry smoothie (control: sugar matched jelly)	348 mg	= SBP, =DBP
Del Bo [28]	Crossover	Controlled	16 smokers, age 24 ± 1, BMI 23 ± 1	Blueberry drink (control: sugar matched drink), followed by smoking 1 cigarette	348 mg	< SBP post smoke, = DBP
Del Bo [29]	Crossover	Controlled	12 healthy male, age 24 ± 1, BMI 23 ± 1	Blueberry drink (control: sugar matched drink)	309 mg	=SBP, =DBP
Del Bo [29]	Crossover	Controlled	12 healthy male smokers, age 15 ± 2, BMI 23 ± 1	Blueberry drink (control: sugar matched drink), followed by smoking 1 cigarette	309 mg	Did not restore blood pressure
Igwe [30]	Crossover	Controlled	12 young (age 31 ± 8, 23 ± 2) and 12 older adults (age 77 ± 6, BMI, 26 ± 3)	Plum juice	369 mg	<SBP, <DBP, <MAP in both age groups, compared to baseline
Keane [31]	Crossover	Single-blind, placebo-controlled	15 male with early hypertension, age 31 ± 9, BMI 27 ± 4	Tart cherry juice	73.5 mg	<SBP, <MAP, = DBP
Keane [32]	Crossover	Double-blind, placebo-controlled	27 healthy, age 50 ± 6, BMI 26 ± 5	Tart cherry juice	68 mg	<SBP
Keane [33]	Crossover	Double-blind, placebo-controlled	10 athletes, age 28 ± 7, BW 78 ± 9	Tart cherry juice	68 mg	<SBP, =DBP, =MAP
Kent [34]	Crossover	Controlled	6 young (age 22 ± 1, BMI 26 ± 4) and 7 older adults (age 78 ± 6, BMI 29 ± 4)	Cherry juice, in single dose or three doses over 2 hours	207 mg	< SBP, <DBP at 2 h after consumption, if given in a single dose (but not if split in three doses given 1 hour apart)
Matsumoto [35]	Crossover	Double-blind, placebo-controlled	9 healthy male, age 30 ± 1, BMI?	Blackcurrant ACN extract	(17 mg/kg)	=SBP, =DBP
Rodriguez-Mateos [36]	Crossover	Double-blind, controlled	10 healthy male, age 27 ± 3, BMI 25 ± 3	Blueberry drink or nutrient-matched control	310 mg	=SBP, =DBP
Rodriguez-Mateos [37]	Crossover	Double-blind, controlled	10 healthy, age 27 ± 1, BMI 25 ± 1	Freeze-dried blueberry drink, blueberry baked product (with same amount of blueberry powder), or baked control	339 mg in drink, 196 mg in baked product	=SBP, =DBP

^a^ Age (in years) and BMI (in kg/m^2^) data expressed as mean ± SD. When data was reported as SEM, SD was calculated as SEM*SQRTparticipant. When BMI was unreported, weight in kg is reported instead. A question mark (?) indicates unreported data. ^b^ When daily ACN dose is unreported, the best alternative information is reported in parentheses. ^c^ Only blood pressure data is reported in this table. Other outcomes of the studies are not reported. SBP, systolic blood pressure; DPB, diastolic blood pressure; MAP, mean arterial pressure; > or <, statistically significant increase or decrease; =, no significant change.

**Table 2:** Summary of long-term interventions.

Reference	Duration	Study Design	Control	Participants ^a^	Source of ACN	Daily Dose of ACN ^b^	Effect on Blood Pressure ^c^
Ataie-Jafari [38]	6 weeks	Pre-post	-	19 women with diabetes, age 53 ± 9, BMI 30 ± 4	Sour cherry juice	720 mg	<SBP, <DBP
Barona [39]	4 weeks	Crossover	Double-blind, placebo-controlled	24 male with, age 51 ± 10, BMI 32 ± 5	Freeze-dried grape phenolic extract	35 mg	<SBP, =DBP
Basu [40]	8 weeks	Parallel arms	Controlled	48 with MetS, age 50 ± 3, BMI 38 ± 2	Freeze-dried blueberry beverage (control: water)	742 mg	<SBP, <DBP
Broncel [41]	8 weeks	Pre-post	-	25 with MetS, age 42–65, BMI 31 ± 3	Aronia extract	300 mg	<SBP, <DBP
Chai [42]	12 weeks	Parallel arms	Controlled	34 overweight older adults, age 70 ± 4, BMI 28 ± 4	Tart cherry juice (control: energy and sugar matched drink)	(451 mg total phenolics)	< SBP, =DBP
Cook [43]	12 days	Crossover	Controlled	15 athletes, age 38 ± 12, BW 76 ± 10	Blackcurrant extract (control: no extract)	105, 210, or 315 mg	= SBP, =DBP, <MAP with 210 and 315
Cook [44]	1 week	Crossover	Double-blind, placebo-controlled	13 healthy male, age 26 ± 4, BMI 25 ± 3	Blackcurrant extract	315 mg	= SBP, =DBP, =MAP at rest, <SBP, <DBP, <MAP during isomeric contraction
Curtis [45]	12 weeks	Parallel arms	Double-blind, placebo-controlled	52 healthy postmenopausal women, age 58 ± 6, BMI 25 ± 4	ACN-rich elderberry extract capsule	500 mg	= SBP, =DBP
Davinelli [46]	4 weeks	Parallel arms	Double-blind, placebo-controlled	42 overweight, age 45–65, BMI 29 ± 4	ACN-rich maqui berry extract	486 mg	= SBP, =DBP
Desai [47]	20 days	Parallel arms	Single-blind, placebo-controlled	11 healthy, age 30 ± 10, BMI 24 ± 3	Tart cherry juice	540 mg	= SBP, =DBP pre or post exercise
Draijer [48]	4 weeks	Crossover	Double-blind, placebo-controlled	60 mildly hypertensive, age 58 ± 10, BMI 26 ± 4	Grape and grape wine extracts	(800 mg total phenolics)	<SBP, <DBP with grape wine but not grape alone
Erlund [49]	8 weeks	Parallel arms	Single-blind, placebo-controlled	72 with CVD risk factors, age 58 ± 6, BMI 26 ± 3	Berry mix (bilberries, lingonberries, black currant, strawberry, chokeberry, and raspberry)	515 mg	<SBP, =DBP
Gurrola-Diaz [50]	4 weeks	Parallel arms	Controlled	73 healthy and 51 MetS patients, age 49 ± 7, BMI 29 ± 5	Hibiscus sabdariffa extract powder (control: preventive diet)	19 mg	= SBP, =DBP in healthy and MetS patients
Habanova [51]	6 weeks	Pre-post	-	36 healthy, age 48 ± 6, BMI 27 ± 4	Frozen bilberries, 3 times a week	456 mg, 3 times/week	= SBP, =DBP
Hassellund [52]	4 weeks	Crossover	Double-blind, placebo-controlled	31 moderately hypertensive male, age 41 ± 3, BMI 27 ± 3	ACN capsule (isolated from bilberry and blackcurrant)	640 mg	= sitting, supine, or 24h-ambulatory blood pressure, or blood pressure during stress test
Jeong [53]	8 weeks	Parallel arms	Double-blind, placebo-controlled	45 prehypertensive, age 57 ± 12, BMI 25 ± 3	Dried unripe black raspberry powder	(1500 mg or 2500 mg powder)	<SBP with high dose, =DBP
Jeong [54]	12 weeks	Parallel arms	Double-blind, placebo-controlled	51 with MetS, age 59 ± 10, BMI 25 ± 4	Dried unripe black raspberry powder	(750 mg of dry powder)	=SBP, =DBP
Johnson [55]	8 weeks	Parallel arms	Double-blind, placebo-controlled	48 postmenopausal women with pre- and stage 1-hypertension, age 59 ± 5, BMI 31 ± 6	Freeze-dried blueberry powder mixed with water	469 mg	<SBP, <DBP
Kardum [56]	4 weeks	Pre-post	-	20 abdominally obese postmenopausal women, age 53 ± 5, BMI 36 ± 4	Glucomannan-enriched aronia juice-based supplement	25 mg	<SBP, =DBP
Kardum [57]	12 weeks	Pre-post	-	29 healthy women, age 35 ± 8, BMI 23 ± 4	Glucomannan-enriched aronia juice-based supplement	25 mg	= SBP, =DBP
Kardum [58]	4 weeks	Pre-post	-	23 pre- or stage 1 hypertensive, age 48 ± 10, weight 82 ± 20	Aronia juice	358 mg	<SBP, <DBP, < average 24 h BP
Karlsen [59]	3 weeks	Parallel arms	Placebo-controlled	118 adults, age 61 ±?, BMI 25 ±?	Purified ACN capsule from bilberry and blackcurrant	300 mg	=SBP, =DBP
Kelley [60]	4 weeks	Pre-post	-	18 healthy, age 50 ± 4, BMI 26 ± 4	Fresh sweet cherries	(280 g fresh cherries)	=SBP, =DBP at the end of the trial and after 1 month
Kent [61]	12 weeks	Parallel arms	Controlled	49 older adults, age 80 ± 6, BMI 26 ± 3	Cherry juice (control: ACN free apple juice)	138 mg	<SBP, =DBP
Khan [62]	6 weeks	Parallel arms	Placebo-controlled	66 healthy adults, age 52 ± 10, BMI 29 ± 6	Blackcurrant juice, low or high dose	40 mg or 143 mg	=SBP, =DBP
Kolehmanen [63]	8 weeks	Parallel arms	Controlled	27 with MetS, age 51 ± 6, BMI 32 ± 4	Bilberries (400 g fresh)	1381 mg	=SBP, =DBP
Lehtonen [64]	20 weeks	Parallel arms	Controlled	61 women, age 43 ±?, BMI 29 ±?	163 g mix of 18 berries (control: lifestyle intervention)	(equivalent to 163 g fresh berries)	=SBP, =DBP
Lee [65]	8 weeks	Parallel arms	Double-blind, placebo-controlled	63 overweight or obese, age 31 ± 9, BMI 28 ± 2	ACN-rich black soybean extract	31.45 mg	=SBP, =DBP
Li [66]	24 weeks	Parallel arms	Double-blind, placebo-controlled	58 with type II diabetes, age 58 ± 3, BMI 24 ± 3,	ACN capsules (isolated from bilberry and black currant)	320 mg	<SBP, =DBP
Loo [67]	16 weeks	Crossover	Single-blind, placebo-controlled	37 with mild hypertension, age 40–70, BMI 26 ± 3	Cold-pressed Aronia juice and oven-dried Aronia powder	1024 mg	<daytime DBP (recorded over 15 hours), =SBP
Lynn [68]	6 weeks	Parallel arms	Controlled	47 healthy adults, age 38 ± 6, BMI 24 ± 3	Tart cherry concentrate, (control: energy matched drink)	274.5 mg	=SBP, =DBP
Matsumoto [35]	2 weeks	Crossover	Double-blind, placebo-controlled	11 healthy, age 39 ± 12, BMI?	Blackcurrant ACN extract	(7.7 mg/kg)	=SBP, =DBP after 30 minutes typing workload
McAnulty [69]	3 weeks	Parallel arms	Controlled	20 smokers, age 28 ± 4, BMI 29 ± 3	Blueberry 250 g		=SBP, =DBP, = ACE activity
McAnulty [70]	6 weeks	Parallel arms	Placebo-controlled	25 healthy, age 43 ± 12, BMI 26 ± 4	Freeze-dried blueberry powder, equivalent to 250 g berries		<aortic systolic pressures, <SBP, =DBP, <DBP in the subset of prehypertensive subjects (9 subjects)
Naruszewicz [71]	6 weeks	Parallel arms	Double-blind, placebo-controlled	44 post myocardial infarction patients, receiving statin therapy, age 66 ± 8, BMI 27 ± 3	255 mg/day Aronia flavonoid extract	64 mg	<SBP, <DBP
Nilsson [72]	5 weeks	Crossover	Controlled	40 healthy, age 63 ± 1, BMI 24 ± 1	Mixed berry drink (1/3 blueberries, 1/9 blackcurrant, 1/9 elderberry, 1/9 lingonberry, 1/9 strawberry, 2/9 tomato), or sugar-matched control	248 mg	=SBP, =DBP
Ohguro [73]	4 weeks	Crossover	Double-blind, placebo-controlled	12 healthy, age 39 ± 8, BMI?	Blackcurrant ACN extract	50 mg	< intraocular pressure, =SBP, =DBP
Ohguro [74]	2 years	Parallel arms	Double-blind, placebo-controlled	21 glaucoma patients, age 61 ± 7, BMI?	Blackcurrant ACN extract	50 mg	< intraocular pressure, =SBP, =DBP
Qin [75]	12 weeks	Parallel arms	Double-blind, placebo-controlled	120 dyslipidemic, age 55 ± 5, BMI 26 ± 4	ACN capsule (from bilberry and blackcurrant)	160 mg	=SBP, =DBP
Puupponen-Pimia [76]	8 weeks	Parallel arms	Controlled	37 overweight with MetS, age 51 ± 7, BMI 32 ± 4	300 g frozen berries (raspberries, strawberries, and cloudberries)	(equivalent to 300 g fresh berries)	=SBP, =DBP
Riso [77]	6 weeks	Crossover	Placebo-controlled	18 male with risk factors for CVD, age 48 ± 10, BMI 25 ± 3	Wild blueberry drink	400 mg	=SBP, =DBP
Skoczynska [78]	6 weeks	Pre-post	-	58 male with mild hypercholesterolemia, age 54 ± 6, BMI 28 ± 3	Aronia juice 250 mL	90 mg	<SBP after 12 weeks, <DBP after 6 and 12 weeks
Sikora [79]	8 weeks	Pre-post	-	23 with untreated MetS (BMI 31 ± 4), reference group with 25 treated MetS patients (BMI 29 ± 3) and 20 healthy controls (BMI 23 ± 1)	Aronia extract supplements or ACE-inhibitors	60 mg	<SBP, <DBP, <ACE activity
Stull [80]	6 weeks	Parallel arms	Double-blind, placebo-controlled	32 obese with insulin-resistance, age 52 ± 3, BMI 37 ± 1	Blueberry powder added to smoothie smoothie and yogurt	580 mg	=SBP, =DBP
Stull [81]	6 weeks	Parallel arms	Double-blind, placebo-controlled	44 adults, age 57 ± 2, BMI 36 ± 1	Blueberry powder added to smoothie and yogurt	580 mg	=SBP, =DBP
Thompson [82]	4 weeks	Crossover	Double-blind, placebo-controlled	16 sedentary, age 38 ± 12, BMI 23 ± 2	ACN capsule	320 mg	=SBP, =DBP
Tjelle [83]	12 weeks	Parallel arms	Double-blind, placebo-controlled	134 prehypertensive or hypertensive, age 52 ± 6, BMI 26 ± 3	Mixed berry fruit juice (red grape, aronia, cherry, and bilberry) or same juice enriched with black currant press residue	43 mg or 210 mg	<SBP in both juice groups, more pronounced if high BP baseline value, =DBP
Udani [84]	4 weeks	Pre-post	-	10 overweight, age 28 ±?, BMI 27 ± 2	100 g açai pulp	0.77 mg/mL ACN (density unknown)	=SBP, =DBP
Whyte [85]	6 months	Parallel arms	Double-blind, placebo-controlled	122 older adults, age 71 ± 4, BW 71 ± 4	Whole wild blueberry powder 1 or 2 g, or extract 200 mg	2.7, or 5.4 or 14 mg	<SBP with extract, but not with powders, at 3 and 6 months
Xie [86]	12 weeks	Parallel arms	Placebo-controlled	49 healthy former smokers, age 35 ± 3, BMI 26 ± 1	500 mg aronia extract	45.1 mg	=SBP, =DBP
Zhang [87]	12 weeks	Parallel arms	Double-blind, placebo-controlled	72 patients with nonalcoholic fatty liver disease, age 46 ± 8, BMI 27 ± 3	ACN capsules (isolated from bilberry and black currant)	320 mg	=SBP, =DBP
Zhu [88]	24 weeks	Parallel arms	Double-blind, placebo-controlled	146 hypercholesterolemic, age 56 ± 6, BMI 27 ± 2	ACN capsules (isolated from bilberry and black currant)	320 mg	=SBP, =DBP

^a^ Age (in years) and BMI (in kg/m^2^) data expressed as mean ± SD. When data was reported as SEM, SD was calculated as SEM*SQRTparticipant. When BMI was unreported, weight in kg is reported instead. A question mark (?) indicates unreported data. ^b^ When daily ACN dose is unreported, the best alternative information is reported in parentheses. ^c^ Only blood pressure data is reported in this table. Other outcomes of the studies are not reported. SBP: systolic blood pressure; DPB: diastolic blood pressure; MetS: Metabolic Syndrome; MAP: mean arterial pressure; ACE: angiotensin-converting enzyme; > or <: statistically significant increase or decrease; =: no significant change.
